# Biopolymer-Based Formulations of *Beauveria bassiana* for Biological Control of the Cabbage Whitefly (*Aleyrodes proletella*)

**DOI:** 10.3390/pathogens15050524

**Published:** 2026-05-13

**Authors:** Mariya Spasova, Emiliya Chervenkova, Atanaska Stoeva, Mariana Petkova, Olya Stoilova

**Affiliations:** 1 Laboratory of Bioactive Polymers, Institute of Polymers, Bulgarian Academy of Sciences, 1113 Sofia, Bulgaria; mspasova@polymer.bas.bg; 2 Department of Entomology, Faculty of Plant Protection and Agroecology, Agricultural University Plovdiv, 4000 Plovdiv, Bulgaria; emilia_chervenkova@au-plovdiv.bg (E.C.); astoeva@au-plovdiv.bg (A.S.); 3 Department of Microbiology and Environmental Biotechnology, Faculty of Plant Protection and Agroecology, Agricultural University Plovdiv, 4000 Plovdiv, Bulgaria; mpetkova@au-plovdiv.bg

**Keywords:** *Beauveria bassiana*, chitooligosaccharide, 2-hydroxyethyl cellulose, *Aleyrodes proletella*, biopolymer formulation, integrated pest management, biological control

## Abstract

Whiteflies, including *Aleyrodes proletella*, are major agricultural pests which cause significant yield losses through direct feeding damage and virus transmission. The entomopathogenic fungus *Beauveria bassiana* is a promising alternative to synthetic insecticides. However, its field performance is often constrained by environmental sensitivity and limited formulation stability. In this study, biopolymer-based suspensions incorporating *B. bassiana* strain 730 were developed using chitooligosaccharide (COS) and 2-hydroxyethyl cellulose (HEC) as biodegradable carriers. Rheological analysis showed increased viscosity upon fungal incorporation (from 75 to 226 cP for COS and from 250 to 354 cP for HEC), indicating effective interaction between the polymer matrices and fungal conidia. Scanning electron microscopy confirmed uniform dispersion and physical entrapment of fungal structures, while microbiological assays demonstrated preserved viability and sporulation capacity. Bioassays against eggs and nymphs of *A. proletella* revealed a clear time-dependent response, with limited efficacy after 24 h but substantial increases by day 3. The unformulated fungal suspension achieved 93.0% efficacy, while COS/*B. bassiana* and HEC/*B. bassiana* formulations reached 84.6% and 76.1%, respectively, comparable to the commercial product Naturalis^®^ (87.2%). Polymer solutions applied alone exhibited significantly lower activity. These results demonstrate that biopolymer-based formulations, particularly COS-based systems, preserve fungal virulence and represent promising biodegradable delivery platforms for sustainable whitefly management.

## 1. Introduction

Whiteflies (Hemiptera: Aleyrodidae) are among the most economically important sap-feeding insect pests affecting both greenhouse and open-field crop production systems. Their high reproductive potential, short life cycle, and efficient phloem-feeding behavior enable rapid population expansion and substantial reductions in plant productivity [1,2]. In addition to direct damage through the removal of photoassimilates, whiteflies excrete honeydew, which promotes the growth of sooty mold, thereby impairing photosynthesis and reducing the market quality of agricultural produce [3]. Certain species, particularly *Bemisia tabaci*, are also recognized as major vectors of plant viruses, further amplifying their economic impact [4]. In temperate regions, the cabbage whitefly, *Aleyrodes proletella*, has emerged as a serious pest of *Brassicaceae* crops, where heavy infestations can lead to significant yield and quality losses [5].

Management of whitefly populations remains challenging due to the widespread development of resistance to conventional insecticides following repeated applications [6]. Moreover, the use of broad-spectrum chemical agents can adversely affect natural enemies and beneficial arthropods, thereby disrupting ecological balance and reducing the effectiveness of integrated pest management strategies [7]. These limitations have stimulated increasing interest in environmentally sustainable alternatives, including microbial biological control agents [8].

Entomopathogenic fungi represent a particularly promising group of microbial control agents, as they infect insect hosts through direct contact and cuticular penetration, offering a mode of action distinct from that of conventional neurotoxic insecticides [9]. Among them, *Beauveria bassiana* (Bals.-Criv.) Vuill. is one of the most extensively studied and commercially applied species, characterized by a broad host range and well-documented efficacy against hemipteran pests. The infection process involves conidial adhesion to the insect cuticle, followed by germination and enzymatic penetration mediated by proteases, chitinases, and lipases [10]. Once inside the host, the fungus proliferates within the hemocoel, leading to systemic colonization and eventual host death, often accompanied by the production of secondary metabolites that contribute to virulence [11].

Despite its considerable potential, the field performance of *B. bassiana* is often inconsistent due to sensitivity to environmental factors such as ultraviolet radiation, temperature fluctuations, and low humidity [12]. In addition, formulation-related challenges, including limited adhesion and persistence on plant surfaces, can reduce conidial survival and infection efficiency [13]. Improving the stability, retention, and delivery of fungal propagules therefore remains a critical step toward enhancing the reliability of fungal biopesticides. Therefore, the development of stable and effective formulations remains a key challenge in the practical application of entomopathogenic fungi for biological control. In this context, polysaccharide-based materials are increasingly explored as environmentally safe formulation agents capable of improving spray characteristics, surface coverage, and retention without introducing additional toxicity [14,15].

Chitosan-derived materials, including chitooligosaccharides (COSs), are particularly attractive due to their film-forming, bioadhesive, and biocompatible properties, which may enhance the persistence of microbial propagules and promote contact with target insects [16,17]. Similarly, 2-hydroxyethyl cellulose (HEC) is a water-soluble modified polysaccharide capable of forming stable and viscous aqueous systems, and has been investigated as a carrier in controlled-release applications, suggesting potential for use in microbial pesticide formulations [18,19]. Previous studies have also demonstrated that COS- and HEC-based coatings can improve surface wettability and support microbial immobilization, contributing to enhanced cell adhesion and viability in biocontrol-related systems [20].

In this context, the present study aims to evaluate the effectiveness of COS- and HEC-based formulations of *Beauveria bassiana* against early developmental stages (eggs and nymphs) of the cabbage whitefly, *Aleyrodes proletella*, and to assess their potential as biopolymer-based delivery systems for fungal biocontrol agents. The experiments were conducted using freshly prepared formulations under controlled laboratory conditions. Further research is required to evaluate long-term storage stability, conidial viability during storage, and formulation performance under field-relevant environmental conditions.

## 2. Materials and Methods

### 2.1. Materials

Chitooligosaccharide (COS; molecular weight 3000–5000 g/mol) was obtained from Kitto Life Co., Ltd. (Pyeongtaek-si, Gyeonggi-do, Republic of Korea). 2-Hydroxyethyl cellulose (HEC; average molecular weight ~90,000 g/mol) was purchased from Sigma-Aldrich (Darmstadt, Germany). Disposable laboratory consumables were supplied by Orange Scientific (Braine-l’Alleud, Belgium).

The entomopathogenic fungal strain *Beauveria bassiana* 730 was provided by Prof. Slavimira Draganova (Agricultural Academy of Bulgaria, Institute of Soil Science, Agro-technologies and Plant Protection, ISSAPP). The strain was originally isolated from *Leptinotarsa decemlineata* (Coleoptera: Chrysomelidae). Fungal cultures were maintained on Potato Dextrose Agar (PDA) and incubated in the dark at 27 °C until conidial formation. Conidia were harvested in dry form and used for subsequent experiments.

A working aqueous conidial suspension was prepared at a concentration of 0.1%, corresponding to approximately 1 × 10^7^ conidia mL^−1^, representing a field-relevant application level. The commercial mycoinsecticide Naturalis^®^ (Biogard, Grassobbio, Italy), based on *B. bassiana*, was included as a reference formulation for comparative evaluation under identical experimental conditions. The selected concentration corresponded to the recommended practical dose of the reference product Naturalis^®^. Future studies should evaluate dose–response relationships to determine concentration-dependent performance of the tested formulations.

Eggs, nymphs, and adults of *Aleyrodes proletella* were collected from experimental fields at the Agricultural University of Plovdiv (Plovdiv, Bulgaria). The collected insects were transferred to the laboratory and maintained on host plant material under controlled environmental conditions until use in bioassays. Prior to treatment, individuals were sorted and assigned to experimental groups corresponding to the different treatment variants.

### 2.2. Preparation of Polymer Solutions and Biohybrid Suspensions

Polymer solutions were prepared at a concentration of 1% (*w*/*v*) by dissolving COS or HEC in distilled water under continuous magnetic stirring at room temperature until complete dissolution. Sterile polymer solutions were aliquoted into 100 mL portions in sterile containers. Subsequently, 50 mL of freshly prepared *B. bassiana* conidial suspension was added to each polymer solution and mixed thoroughly to ensure uniform dispersion of fungal propagules within the matrix. The resulting mixtures were used as biohybrid suspensions.

### 2.3. Characterization

The dynamic viscosity of polymer solutions and suspensions was measured using a Brookfield DV-II+ Pro viscometer (Brookfield Engineering Laboratories, Middleboro, MA, USA) equipped with a cone spindle (CPE-52) at 25 °C.

The surface morphology of polymer films and *B. bassiana* structures was examined by scanning electron microscopy (SEM) using a JSM-5510 instrument (JEOL Co., Ltd., Tokyo, Japan). Thin films were prepared by casting the polymer solutions, *B. bassiana* suspensions, and biohybrid formulations onto aluminum foil and allowing them to air-dry at room temperature. Dried samples were mounted on SEM stubs using double-sided conductive carbon tape and coated with a thin gold layer using a JEOL JFC-1200 sputter coater (JEOL Co., Ltd., Tokyo, Japan) for 120 s at 20 mA. Imaging was performed under high vacuum at an accelerating voltage of 10 kV. Micrographs were recorded at different magnifications to evaluate film structure and fungal distribution within the matrices.

Microscopic observations of insect specimens were performed using a Zeiss Stemi 2000-C stereomicroscope (Carl Zeiss AG, Oberkochen, Germany) equipped with a Zeiss AxioCam ERc5s digital camera.

### 2.4. Fungal Growth Assay

To assess the effect of polymer incorporation on fungal viability, growth assays were performed on yeast extract agar (YEA; Merck, Darmstadt, Germany). The medium consisted of peptically digested animal tissue (5.0 g/L), yeast extract (3.0 g/L), and agar (15.0 g/L), and was prepared according to standard microbiological protocols.

After sterilization, the medium was poured into sterile Petri dishes and allowed to solidify. Aliquots (10 µL) of each biohybrid suspension were applied to the center of the agar surface. Polymer solutions without fungal inoculum were included as controls.

Each treatment was tested in five biological replicates (*n* = 5). Plates were incubated at 25 °C for five days. Colony growth was monitored at 24, 48, 72, and 120 h. Colony diameter was determined as the mean of two perpendicular measurements. Sporulation was evaluated qualitatively based on visible conidial formation.

### 2.5. Experimental Design and Treatment Structure

A laboratory bioassay was conducted to evaluate the efficacy of *Beauveria bassiana* strain 730 applied either alone or in combination with polymer carriers. The experimental design followed standard approaches for evaluating microbial biocontrol agents.

Seven treatments were evaluated: distilled water was used as a blank control to establish baseline mortality and enable correction of efficacy; the fungal treatment consisted of *B. bassiana* strain 730 applied at a field-relevant concentration of 0.1%; to assess the independent effects of the carrier systems, COS and HEC were tested individually as polymer controls; two biohybrid formulations were prepared by combining *B. bassiana* with COS or HEC, respectively, to evaluate the performance of immobilized fungal systems; and the commercial product Naturalis^®^ (0.1%), based on *B. bassiana*, was included as a positive reference control for comparative assessment of efficacy.

Conidia from actively growing cultures were suspended in sterile distilled water containing a small amount of surfactant to improve dispersion. The suspension was adjusted to approximately 1 × 10^7^ conidia mL^−1^. For combined treatments, the fungal suspension was mixed with the respective polymer solution immediately before application. The final fungal concentration was maintained at the same level in all fungal-containing treatments. All suspensions were freshly prepared and gently agitated prior to use.

### 2.6. Bioassay Conditions

All experiments were performed under controlled laboratory conditions at 25 ± 1 °C, relative humidity of approximately 70%, and a 16:8 h light: dark photoperiod.

Adult whiteflies *A. proletella* from a laboratory-maintained colony were allowed to oviposit on cabbage plants bearing 5–6 fully developed leaves (approximately 20 adults per plant). Plants were kept in plexiglass cages (20 × 20 × 60 cm) covered with fine mesh. After 48 h, the adults were removed.

Detached leaves containing eggs and nymphs were used as experimental units. The number of individuals per leaf was recorded before treatment and ranged from approximately 10 to 50 eggs or nymphs, reflecting natural variation in infestation density. Each treatment consisted of three independent biological replicates (n = 3), corresponding to approximately 210–240 individuals per treatment.

Formulations were applied using a hand-held sprayer, delivering 1 mL per replicate to obtain uniform coverage of the leaf surface and associated insect stages. Control leaves received distilled water only. After treatment, leaves were air-dried and transferred to Petri dishes containing moist filter paper to maintain humidity during incubation.

### 2.7. Mortality Assessment and Corrected Efficacy

Live and dead individuals of *Aleyrodes proletella* were recorded separately for eggs and nymphs on day 0 (before treatment), day 1, and day 3 after application. Eggs were considered non-viable when they appeared collapsed, desiccated, discolored, failed to hatch by day 3, or showed visible fungal colonization. Nymphs were considered dead when no movement was observed after gentle stimulation together with visible signs of loss of viability. Because egg hatch and stage transition occurred during the observation period, mortality was assessed separately for eggs and nymphs to avoid bias caused by ontogenetic transitions.

For the egg stage, individuals were classified into three categories: live eggs, dead eggs, and hatched nymphs. Since reductions in live egg counts may result from either mortality or hatching, the number of hatched nymphs was estimated using a mass-balance approach adapted from Boukouvala et al. [21]:

Hatched nymphs = Initial eggs − (Live eggs + Dead eggs)(1)

Thus:

Initial eggs = Live eggs + Dead eggs + Hatched nymphs(2)

Egg mortality (%) was calculated as the proportion of dead eggs relative to the total number of unhatched eggs at each observation time:

Egg mortality (%) = Dead eggs/(Live eggs + Dead eggs) × 100(3)

Hatched nymphs represent successfully developed individuals and were excluded from mortality calculations.

For nymphs, mortality (%) was calculated as:

Nymph mortality (%) = Dead nymphs/(Live nymphs + Dead nymphs) × 100(4)

To avoid biologically ambiguous interpretation caused by stage transitions, corrected efficacy was not calculated for stage-specific data. Instead, treatment performance was evaluated only for the combined population (eggs and nymphs pooled) using the Henderson–Tilton formula:

(5) $$E,\%=(1-\left(\frac{T_{a}}{T_{b}}\right)\times \left(\frac{C_{b}}{C_{a}}\right))\times 100,$$

where *T_b_* and *T_a_* are the numbers of living individuals in treated units before and after treatment, respectively, and *C_b_* and *C_a_* are the corresponding values in the control. Because naturally infested leaves differed in initial population density, all calculations were based on replicate-specific baseline counts.

### 2.8. Statistical Analysis

All experimental data are presented as mean ± standard deviation (SD) of three independent biological replicates (*n* = 3). Differences among treatments at each observation time were analyzed by one-way analysis of variance (ANOVA), followed by Tukey’s honestly significant difference (HSD) test when significant effects were detected (*p* < 0.05). Normality and homogeneity of variance were evaluated using the Shapiro–Wilk and Levene’s tests, respectively. Percentage data were arcsine square-root transformed when necessary to satisfy ANOVA assumptions; however, non-transformed means are presented in the tables for clarity.

## 3. Results

### 3.1. Rheological Properties and SEM Characterization

The rheological behavior of the polymer systems was evaluated to assess their suitability as formulation matrices. The dynamic viscosities of 1% (*w*/*v*) aqueous solutions were 75 cP for chitooligosaccharide (COS) and 250 cP for 2-hydroxyethyl cellulose (HEC). The higher viscosity of HEC is attributed to its higher molecular weight and greater chain entanglement. Incorporation of *Beauveria bassiana* significantly increased viscosity, reaching 226 cP for COS-based suspensions and 354 cP for HEC-based suspensions, indicating interactions between fungal propagules and the polymer networks.

**Figure 1:**
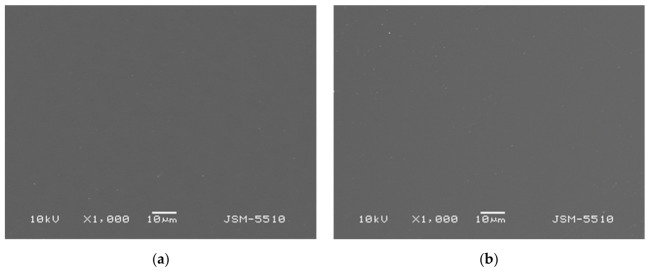

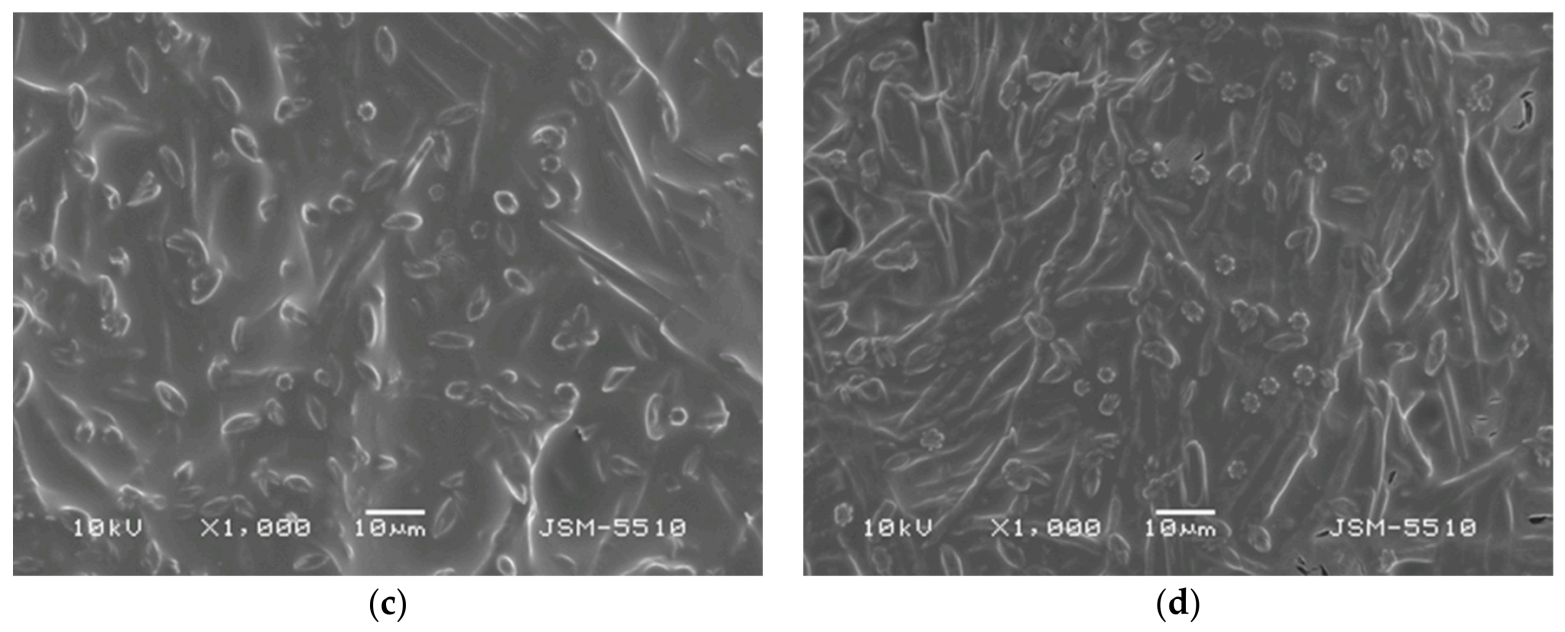
Scanning electron micrographs of films prepared by casting: (**a**) COS solution, (**b**) HEC solution, (**c**) COS/*B. bassiana* suspension, and (**d**) HEC/*B. bassiana* suspension.

Because the formulations were applied as liquid suspensions, thin films were prepared by casting and drying to enable morphological analysis by scanning electron microscopy (SEM). SEM was intended only to confirm physical incorporation of fungal structures, morphology retention and dispersion within the polymer matrix. Representative micrographs are shown in Figure 1. Films obtained from both COS and HEC were smooth, homogeneous, and free of structural defects, confirming good film-forming capacity (Figure 1a,b). In the presence of *B. bassiana*, fungal structures were clearly visible within the polymer matrices (Figure 1c,d). Hyphae appeared as elongated filamentous structures, while conidia were observed as small, oval to sub-spherical units distributed throughout the films. Fungal elements retained their characteristic morphology and were uniformly dispersed without evidence of aggregation. The polymer phase formed a continuous matrix embedding or partially covering the fungal structures, confirming successful physical immobilization. These observations demonstrate effective incorporation of *B. bassiana* into both COS and HEC systems. SEM analysis confirmed the successful physical incorporation and distribution of fungal propagules within the polymer matrices, but it does not by itself demonstrate functional superiority. For comparative purposes, the morphology of unformulated *B. bassiana* is also presented (Figure S1, Supplementary Material).

### 3.2. Fungal Viability After Polymer Incorporation

**Figure 2:**
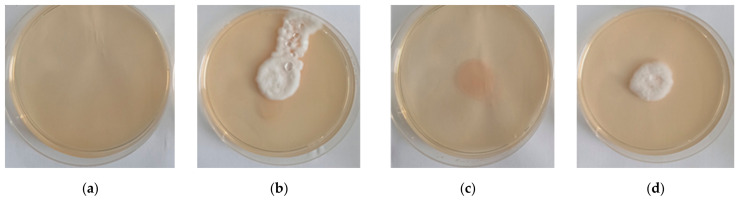
Growth of *B. bassiana* on yeast extract agar after 24 h: (**a**) COS solution (sterility control), (**b**) COS/*B. bassiana* suspension, (**c**) HEC solution (sterility control), and (**d**) HEC/*B. bassiana* suspension.

The effect of polymer incorporation on fungal viability was assessed using a yeast extract agar assay (Figure 2). No microbial growth was detected in polymer-only controls (COS and HEC), confirming sterility (Figure 2a,c). In contrast, both COS/*B. bassiana* and HEC/*B. bassiana* suspensions exhibited visible radial growth within 24 h at 25 °C, with continued colony expansion over the five-day observation period (Figure 2b,d). Differences in growth characteristics were observed between formulations. Colonies derived from COS-based suspensions showed greater radial expansion, denser mycelial networks, and more pronounced sporulation compared with those obtained from HEC-based systems. These differences are likely related to polymer physicochemical properties. Lower-molecular-weight COS may form more permeable matrices, facilitating diffusion of water and nutrients and promoting fungal growth. Importantly, fungal development occurred in both systems, indicating that polymer incorporation did not inhibit viability or vegetative growth. Although unformulated *B. bassiana* conidia were included as a reference treatment in the bioassay experiments, they were not included as a separate control in the agar growth assay presented in this section. Therefore, the current assay primarily demonstrates that fungal propagules remained viable and capable of growth after incorporation into the polymer matrices, rather than providing a direct comparison with unformulated conidia.

### 3.3. Microscopic Assessment of Aleyrodes proletella

**Figure 3:**
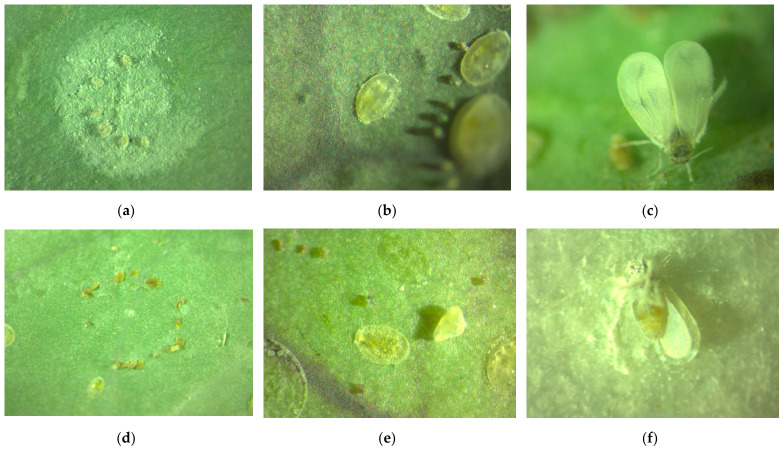
Microscopic images of *A. proletella* before (**a**–**c**) and after treatment (**d**–**f**) with *B. bassiana*: (**a**) viable egg; (**b**) healthy nymph; (**c**) healthy adult; (**d**) collapsed egg; (**e**) infected nymph; (**f**) adult with external fungal growth. Magnification ×10.

Microscopic examination revealed clear morphological differences between untreated individuals and those exposed to *B. bassiana* (Figure 3). Control eggs exhibited a typical oval shape with smooth chorion and translucent internal contents, indicating normal embryonic development (Figure 3a). Nymphs showed transparent cuticles and well-defined internal structures (Figure 3b), while adults displayed intact morphology without signs of microbial colonization (Figure 3c). In contrast, treated specimens showed pronounced alterations across all developmental stages. Eggs appeared collapsed and opaque, indicating loss of internal integrity (Figure 3d). Nymphs exhibited cuticular opacity and deformation consistent with progressive infection (Figure 3e). Adult individuals showed extensive external mycelial growth, confirming successful fungal colonization and post-mortem sporulation (Figure 3f). These observations provide direct visual evidence of infection, tissue degradation, and fungal proliferation in *A. proletella*.

### 3.4. Stage-Specific Population Dynamics and Corrected Efficacy Against Aleyrodes proletella

Under control conditions, a decrease in the number of live eggs accompanied by a corresponding increase in nymphs by day 3 confirmed normal egg hatch and stage transition during the observation period. This demonstrates that changes in stage-specific counts reflect both developmental processes and treatment effects. The 3-day observation interval was selected to evaluate the early pathogenic activity of Beauveria bassiana under controlled laboratory conditions. This interval is particularly relevant for *Aleyrodes proletella*, a rapidly developing pest capable of fast population increase.

The egg stage is presented in Table 1. At day 1, egg mortality remained low across all treatments, indicating the early (latent) phase of fungal infection. By day 3, clear treatment effects were observed. The highest egg mortality was recorded for *B. bassiana* alone and COS/*B. bassiana*, both reaching 100% mortality of unhatched eggs. High mortality was also observed for HEC/*B. bassiana* (98.2%). The commercial reference Naturalis^®^ showed moderate activity (56.1%). Polymer-only treatments also resulted in measurable mortality, with COS (82.4%) exceeding HEC (50.0%). However, these effects were lower or more variable compared with fungal-containing treatments and should be interpreted cautiously, as they may reflect indirect or physical effects rather than true ovicidal activity. These results indicate that fungal treatments were the primary drivers of egg mortality, while polymer matrices alone had limited or inconsistent direct effects.

The nymph stage is presented in Table 2. Nymphs responded more rapidly than eggs. At day 1, fungal treatments, particularly Naturalis^®^ (48.7%) and *B. bassiana* (22.0%), already produced measurable mortality compared with the control and polymer-only treatments. By day 3, all fungal-containing treatments resulted in high mortality, with COS/*B. bassiana* (85.1%) and Naturalis^®^ (82.1%) among the most effective. *B. bassiana* alone (75.7%) and HEC/*B. bassiana* (68.2%) also showed strong activity. Polymer-only treatments produced moderate mortality (COS: 49.0%; HEC: 46.2%). Therefore, nymphs exhibited a faster and more pronounced response than eggs, indicating higher susceptibility of this developmental stage under the tested laboratory conditions.

**Figure 4:**
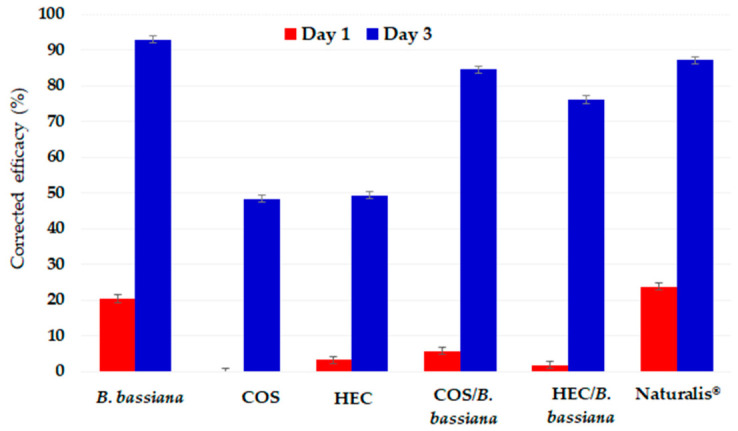
Corrected efficacy (%) of treatments against the total population of *Aleyrodes proletella* (eggs and nymphs combined) at 1 and 3 days after application, calculated using the Henderson–Tilton formula based on pooled live counts. Values are mean ± SD (*n* = 3).

Corrected efficacy based on the combined population (eggs and nymphs pooled) is presented in Figure 4. Efficacy increased markedly from day 1 to day 3 for all fungal-containing treatments, indicating a clear time-dependent response. By day 3, the highest corrected efficacy was observed for *B. bassiana* alone (93.0%), followed by Naturalis^®^ (87.2%), COS/*B. bassiana* (84.6%), and HEC/*B. bassiana* (76.1%). Polymer-only treatments showed substantially lower efficacy.

The obtained results demonstrate strong short-term pathogenicity of strain 730 against early developmental stages of *A. proletella*. Incorporation into COS- and HEC-based matrices retained substantial biological activity. However, freshly applied unformulated conidia showed the highest efficacy under the present laboratory conditions. These findings confirm that fungal pathogenicity was the primary driver of population suppression, while polymer-based matrices preserved biological activity without exceeding the short-term efficacy of the unformulated fungal suspension.

## 4. Discussion

The novelty of the present study lies in the combination of a locally selected virulent isolate, *Beauveria bassiana* strain 730, with two water-soluble biodegradable matrices (COS and HEC), evaluated comparatively under identical conditions against *Aleyrodes proletella*, a pest for which formulation data remain limited. A further advantage is the use of chitooligosaccharide (COS), which, unlike conventional chitosan, is fully water-soluble and does not require acidic solvents that may negatively affect conidial viability or alter spray characteristics. Both COS and HEC are biodegradable and compatible with low-input crop protection strategies, supporting their suitability as environmentally benign formulation components. In addition, the study integrates biological efficacy with physicochemical characterization, showing that incorporation of conidia affects rheological properties, which is relevant for sprayability and formulation behavior. These findings contribute to the development of biodegradable, microplastic-free delivery systems with efficacy approaching that of the commercial product Naturalis^®^ [22,23].

The results confirm that *B. bassiana* strain 730 exhibits strong pathogenic activity against early developmental stages of *A. proletella* under controlled laboratory conditions. Mortality increased markedly between day 1 and day 3 in all fungal-containing treatments, consistent with the known infection cycle of entomopathogenic fungi, which involves conidial adhesion, germination, cuticle penetration, and internal colonization prior to host death. The observed time-dependent increase in efficacy reflects this sequential infection process and is in agreement with previous studies on fungal entomopathogens [24]. The high efficacy of the unformulated fungal suspension confirms the intrinsic virulence of strain 730 and supports its potential as a biological control agent. Its performance was comparable to that of the commercial reference product Naturalis^®^, indicating that locally adapted strains may provide effective alternatives for pest management. However, it is well established that unformulated conidia are highly sensitive to environmental stressors such as ultraviolet radiation, temperature fluctuations, and low humidity, which can significantly reduce persistence and field efficacy [24]. Under laboratory conditions, freshly applied conidia are immediately available for host contact, which is likely to explain their strong short-term performance. In contrast, conidia incorporated into polymer matrices may exhibit modified release or contact dynamics during the short observation period.

Polymer-based formulations containing COS or HEC retained substantial biological activity, demonstrating compatibility of these matrices with fungal viability and infectivity. This is an important finding, as formulation additives can sometimes negatively affect microbial performance. In the present study, incorporation into polymer systems preserved fungal activity and, in several cases, maintained efficacy close to that of the unformulated suspension. This observation is consistent with previous reports that polysaccharide-based materials primarily function as formulation carriers, enhancing delivery, spreading, and retention rather than acting as direct toxic agents [25,26]. Stage-dependent responses were clearly observed. Nymphs responded more rapidly than eggs, while final efficacy after 3 days varied among treatments. Eggs are protected by chorionic layers that may delay fungal penetration, whereas nymphs represent exposed feeding stages that facilitate contact with fungal propagules. Such differences in susceptibility between developmental stages have been reported previously and are relevant for optimizing application timing in pest management programs [24]. Polymer-only treatments also caused measurable effects, particularly in nymphs. Because COS and HEC are not known insecticidal agents, these effects are likely indirect, potentially involving changes in leaf surface properties, moisture retention, or physical interference with insect physiology. However, these mechanisms were not directly investigated in the present study and require further research.

Importantly, incorporation of *B. bassiana* into polymer matrices did not impair fungal infectivity. Both COS- and HEC-based formulations retained high levels of biological activity, supporting the feasibility of matrix-assisted delivery systems. Similar findings have been reported in formulation studies demonstrating that encapsulation or polymer-based carriers can preserve microbial viability while improving handling and application properties [27]. Nevertheless, under the short-term laboratory conditions applied here, none of the formulations exceeded the efficacy of the unformulated suspension. This indicates that, in such conditions, mortality is primarily driven by fungal virulence rather than formulation effects, a pattern also observed in other microbial pesticide studies [28].

Among the tested systems, COS-based formulations generally showed performance closer to that of the unformulated fungus than HEC-based systems. This may be related to physicochemical properties such as film-forming capacity or adhesion to leaf surfaces, which could influence conidial distribution and retention. However, these mechanisms were not directly measured and should be considered as hypotheses for future investigation. A key limitation of this study is that it was conducted under controlled laboratory conditions over a short observation period. Environmental stress factors such as UV radiation, rainfall, and fluctuating temperature were not included, although they are known to affect fungal persistence and efficacy. Consequently, the practical advantages of polymer-based formulations, such as improved protection and retention, ay become more evident under greenhouse or field conditions. Future work should therefore focus on field validation, storage stability, persistence on plant surfaces, and interactions with environmental factors. Such studies will be essential to fully assess the formulation potential of these systems.

## 5. Conclusions

The present study demonstrates that *Beauveria bassiana* strain 730 is a promising biological control candidate against early developmental stages of *Aleyrodes proletella* under laboratory conditions. The fungus induced substantial mortality in both eggs and nymphs within three days after treatment and performed comparably to the commercial reference product Naturalis^®^. Incorporation of fungal conidia into biodegradable polymer matrices based on chitooligosaccharide (COS) and 2-hydroxyethyl cellulose (HEC) did not impair fungal activity. Several formulated variants maintained efficacy levels close to that of the unformulated suspension, indicating that these materials are suitable carrier systems for fungal propagules. Among the tested matrices, COS-based formulations showed the most favorable overall performance. Polymer-only treatments produced moderate effects, suggesting that their primary role is supportive rather than directly insecticidal. The results highlight that fungal virulence is the principal determinant of mortality, whereas formulation strategy influences delivery and application performance. Further research is required to evaluate storage stability, environmental persistence, and field efficacy in order to support the practical implementation of these formulations within integrated pest management programs.

## Figures and Tables

**Table 1:** Stage-specific dynamics of eggs of *Aleyrodes proletella* following treatment with *Beauveria bassiana* and polymer-based formulations at 0, 1, and 3 days after application. Values are mean ± SD (*n* = 3).

Treatment	Day	Alive Eggs	Dead Eggs	Hatched Nymphs	Egg Mortality (%)
Control (water)	0	47.3 ± 2.5	0.0 ± 0.0	0.0 ± 0.0	0.0 ± 0.0
	1	47.3 ± 2.5	0.0 ± 0.0	0.0 ± 0.0	0.0 ± 0.0
	3	32.3 ± 3.1	0.0 ± 0.0	15.0 ± 0.0	0.0 ± 0.0
*Beauveria bassiana*	0	33.7 ± 3.0	0.0 ± 0.0	0.0 ± 0.0	0.0 ± 0.0
	1	27.0 ± 2.6	6.3 ± 1.2	0.3 ± 1.1	19.0 ± 1.3
	3	0.0 ± 0.0	33.3 ± 2.5	0.3 ± 1.4	100.0 ± 0.0
COS solution	0	7.0 ± 2.0	0.0 ± 0.0	0.0 ± 0.0	0.0 ± 0.0
	1	6.3 ± 1.5	0.0 ± 0.0	10.7 ± 2.5	0.0 ± 0.0
	3	1.0 ± 0.6	4.7 ± 1.2	11.3 ± 2.5	82.4 ± 1.4
HEC solution	0	10.3 ± 1.8	0.0 ± 0.0	0.0 ± 0.0	0.0 ± 0.0
	1	9.7 ± 1.5	0.0 ± 0.0	5.6 ± 2.3	0.0 ± 0.0
	3	4.0 ± 1.0	4.0 ± 1.0	7.3 ± 2.1	50.0 ± 1.2
COS/*B. bassiana*	0	31.3 ± 2.5	0.0 ± 0.0	0.0 ± 0.0	0.0 ± 0.0
	1	29.7 ± 2.0	0.0 ± 0.0	1.7 ± 1.5	0.0 ± 0.0
	3	0.0 ± 0.0	24.3 ± 2.2	7.0 ± 2.3	100.0 ± 0.0
HEC/*B. bassiana*	0	38.3 ± 2.8	0.0 ± 0.0	0.0 ± 0.0	0.0 ± 0.0
	1	36.3 ± 2.5	0.0 ± 0.0	2.0 ± 1.7	0.0 ± 0.0
	3	0.7 ± 0.6	36.3 ± 2.8	1.3 ± 1.9	98.2 ± 2.3
Naturalis^®^	0	39.0 ± 3.0	0.0 ± 0.0	0.0 ± 0.0	0.0 ± 0.0
	1	37.7 ± 2.7	1.3 ± 0.5	3.0 ± 1.9	3.4 ± 1.0
	3	15.7 ± 2.2	20.0 ± 2.5	2.3 ± 2.2	56.1 ± 2.1

**Table 2:** Stage-specific dynamics of nymphs of *Aleyrodes proletella* following treatment with *Beauveria bassiana* and polymer-based formulations at 0, 1, and 3 days after application. Values are mean ± SD (*n* = 3).

Treatment	Day	Alive Nymphs	Dead Nymphs	Nymph Mortality (%)
Control (water)	0	32.7 ± 2.1	0.0 ± 0.0	0.0 ± 0.0
	1	32.7 ± 2.1	0.0 ± 0.0	0.0 ± 0.0
	3	32.7 ± 2.3	0.0 ± 0.0	0.0 ± 0.0
*Beauveria bassiana*	0	13.7 ± 1.5	0.0 ± 0.0	0.0 ± 0.0
	1	10.7 ± 1.3	3.0 ± 0.8	22.0 ± 2.0
	3	3.3 ± 0.7	10.3 ± 1.2	75.7 ± 2.5
COS solution	0	34.7 ± 2.4	0.0 ± 0.0	0.0 ± 0.0
	1	34.7 ± 2.2	0.0 ± 0.0	0.0 ± 0.0
	3	17.7 ± 1.8	17.0 ± 1.9	49.0 ± 2.3
HEC solution	0	39.7 ± 2.6	0.0 ± 0.0	0.0 ± 0.0
	1	38.7 ± 2.4	1.0 ± 0.5	2.5 ± 0.9
	3	21.3 ± 1.7	18.3 ± 2.1	46.2 ± 2.4
COS/*B. bassiana*	0	38.0 ± 2.0	0.0 ± 0.0	0.0 ± 0.0
	1	34.3 ± 2.0	3.7 ± 0.9	9.7 ± 1.5
	3	5.7 ± 1.1	32.3 ± 2.5	85.1 ± 2.8
HEC/*B. bassiana*	0	35.7 ± 2.2	0.0 ± 0.0	0.0 ± 0.0
	1	34.7 ± 2.1	1.0 ± 0.5	2.8 ± 1.0
	3	11.3 ± 1.4	24.3 ± 2.2	68.2 ± 2.6
Naturalis^®^	0	13.0 ± 1.6	0.0 ± 0.0	0.0 ± 0.0
	1	6.7 ± 1.1	6.3 ± 1.0	48.7 ± 2.4
	3	2.3 ± 0.6	10.7 ± 1.3	82.1 ± 2.7

## Data Availability

Data are contained within the article.

## Supplementary Materials

The following supporting information can be downloaded at: https://www.mdpi.com/article/10.3390/pathogens15050524/s1, Figure S1: Scanning electron micrograph of *B. bassiana*.

## Abbreviations

The following abbreviations are used in this manuscript:

COS	Chitooligosaccharide
HEC	2-Hydroxyethyl cellulose
