# Supplementary material for: Biopolymer-Based Formulations of Beauveria bassiana for Biological Control of the Cabbage Whitefly (Aleyrodes proletella)
Source: Pathogens. 2026 May 13;15(5):524. doi: 10.3390/pathogens15050524 (PMC13210045; doi:10.3390/pathogens15050524)
Supplement: Supplementary file 1 [file pathogens-15-00524-s001.zip › pathogens-4241179-supplementary.pdf]

Supplementary data

Biopolymer-Based Formulations of *Beauveria bassiana* for Bio-logical Control of the Cabbage Whitefly (*Aleyrodes proletella*)

Mariya Spasova [1], Emiliya Chervenkova [2], Atanaska Stoeva [2], Mariana Petkova [3] and Olya Stoilova [1,*]

[1] Laboratory of Bioactive Polymers, Institute of Polymers, Bulgarian Academy of Sciences, 1113 Sofia, Bulgaria; mspasova@polymer.bas.bg
[2] Department of Entomology, Faculty of Plant Protection and Agroecology, Agricultural University Plovdiv, 4000 Plovdiv, Bulgaria; emilia_chervenkova@au-plovdiv.bg (E.C.); astoeva@au-plovdiv.bg (A.S.)
[3] Department of Microbiology and Environmental Biotechnology, Faculty of Plant Protection and Agroe-cology, Agricultural University Plovdiv, 4000 Plovdiv, Bulgaria; mpetkova@au-plovdiv.bg
* Correspondence: stoilova@polymer.bas.bg

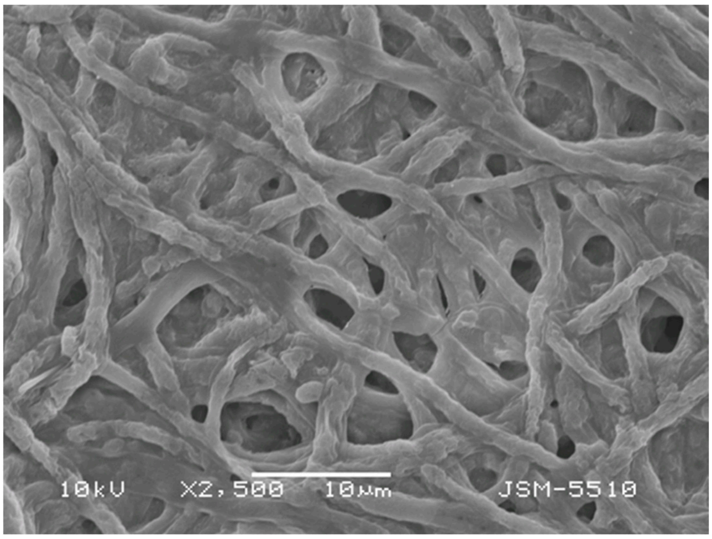

Figure S1. Scanning electron micrograph of *Beauveria bassiana*.

Academic Editor: Giovanni Bubici

Received: 20 March 2026
Revised: 1 May 2026
Accepted: 11 May 2026
Published: 13 May 2026

Copyright: © 2026 by the authors. Submitted for possible open access publication under the terms and conditions of the Creative Commons Attribution (CC BY) license.
